# Tool Wear and Surface Finish in AISI 304 Stainless Steel Dry Turning with Cermet Inserts

**DOI:** 10.3390/ma19061274

**Published:** 2026-03-23

**Authors:** Laurence Colares Magalhães, Nelson Antenor Sorte, Marcelo Tramontin Souza, Armando Marques

**Affiliations:** 1 Department of Industrial Technology, Federal University of Espírito Santo (UFES), Vitoria 29075-910, Brazil; 2 Department of Engineering and Computing, Santa Cruz State University (UESC), Ilheus 45662-900, Brazil; 3 Federal Institute of Espírito Santo, Vitoria 29047-910, Brazil

**Keywords:** dry turning, clean production, green manufacturing, cermet tool wear, AISI 304 stainless steel

## Abstract

The present study investigates the surface integrity and flank wear of uncoated cermet inserts during dry turning of AISI 304 stainless steel. Three-dimensional metrology techniques were employed to assess both surface roughness and cutting-tool flank wear. Cutting speed and feed rate were the process parameters varied in the experiments. Both parameters exhibited a significant influence on the final surface quality. Specifically, increasing the cutting speed resulted in a deterioration of the surface finish under the evaluated conditions. Considering an average flank wear (VBB) of 0.1 mm as the tool life criterion, tool lives of 15 min and 9 min were achieved at cutting speeds of 120 m/min (lowest level) and 150 m/min (highest level), respectively. At lower cutting speeds, abrasive wear and adhesion were the predominant wear mechanisms, whereas chipping and diffusion became more pronounced at the higher cutting speed. The dry turning of AISI 304 stainless steel with uncoated cermet inserts proved viable in terms of sustainability and surface integrity; however, effective chip evacuation remains a critical concern. The use of compressed air or minimum quantity lubrication (MQL) may help mitigate this issue.

## 1. Introduction

Cleaner production has become a central objective in modern manufacturing. Sustainable production refers to practices that do not compromise the needs of future generations, and contrary to common belief, cleaner production does not necessarily require increased financial investment. A major opportunity for reducing environmental impacts in machining lies in minimizing pollution associated with the use of coolants and emulsions. Cleaner production strategies can therefore enhance environmental performance by reducing waste and emissions [1]. Analyses of traditional wet machining have highlighted several drawbacks, reinforcing that dry cutting, an important pillar of green manufacturing, offers advantages such as lower environmental pollution and more rational resource utilization [2].

Machining industries continuously seek high material removal rates and high product quality, typically by employing elevated cutting speeds and feed rates. However, achieving these goals becomes increasingly challenging due to the high cutting temperatures generated in the cutting zone. Excessive heat contributes to premature tool failure, poor dimensional accuracy, and deterioration in surface integrity through tensile residual stresses, microcracks, and accelerated oxidation and corrosion [3]. Residual stresses, in particular, are critical because of their strong influence on the fatigue performance of machined components.

Cooling lubricants play essential roles in machining: they reduce frictional heat generation, protect the subsurface integrity of the workpiece by extracting heat, and assist in chip evacuation. Effective lubrication systems therefore enable high-performance operations [4]. In dry machining, however, the absence of coolant increases friction and adhesion at the tool-workpiece interface, raising thermal loads on both tool and material. These elevated temperatures may accelerate tool wear, such as crater formation when turning steels with uncoated carbides, but can also reduce thermal shock, thereby lowering the tendency for comb crack formation in interrupted cutting with carbides or cermets. Higher machining temperatures also influence chip formation, often promoting the occurrence of ribbon or snarl chips [4].

Despite their functional benefits, cooling lubricants pose environmental and economic challenges. Their chemical degradation at high temperatures generates hazardous waste, and their disposal risks soil and water contamination. Coolant systems also incur high operational costs due to storage, pumping, filtration, and recycling requirements, while posing health and safety concerns to machine operators and causing corrosion in machine components and workpieces [3,4].

Recent advances in cutting tool materials have supported the adoption of dry machining or minimum quantity lubrication (MQL), particularly in steels and alloys. In some cases, dry machining can achieve equal or superior surface quality compared with MQL, and tool wear differences may be relatively small depending on cutting parameters [5]. Cermets are among the most suitable tool materials for dry cutting due to their high hot hardness, low chemical affinity with steels, and lower thermal conductivity relative to cemented carbides [6]. Comparing a typical cemented carbide (WC–6Co) with a TiC-based cermet, Klocke [7] reported average microhardness values of 1580 HV (HV30) and 3100 HV (HV0.05), and thermal conductivities of 80 W/(m·K) and 33 W/(m·K), respectively. Additional characteristics, such as excellent wear resistance, sharp cutting edges, high cutting speed capability under modest feeds and depths of cut, and good surface finish, further contribute to the competitive performance of cermets [8]. Given their favorable cost-performance balance, cermet tools are a strong alternative for semi-finishing and finishing operations in steels and cast irons [9].

Other recent studies have continued to investigate the machining performance and wear behavior of cermet tools. For example, Zhou et al. [10] analyzed the cutting performance and wear mechanisms of ultrafine Ti(C,N)-based cermets during high-speed dry cutting, highlighting the combined effects of abrasive, adhesive, and oxidative wear under severe thermo-mechanical conditions. Wu et al. [11] investigated the wear behavior of cutting tools during machining of AISI 304 stainless steel and reported that high friction, work hardening, and elevated temperatures significantly accelerate tool degradation during machining of this alloy. The comprehensive review by Zhu et al. [12] summarize advances in Ti(C,N)-based cermets, emphasizing their excellent high-temperature mechanical properties and wear resistance, which make them promising candidates for high-performance metal machining applications.

Tool wear and surface roughness are widely recognized as primary indicators of machinability [13], and extensive research has explored the turning of stainless and alloy steels under various cooling conditions and with different tool materials. Also, most studies rely primarily on conventional 2D roughness parameters, whereas three-dimensional surface metrology can provide a more comprehensive description of functional surface characteristics. Kulkarni et al. [14] evaluated the tool life of AlTiCrN-coated inserts in dry turning of AISI 304 stainless steel and found improved surface finish and lower cutting forces at 320 m/min and 0.08 mm/rev, with optimum tool wear between 200 and 260 m/min. Chen et al. [15] studied tool wear mechanisms in dry turning of AISI 304 with cemented carbide inserts at 80 m/min, observing abrasive, adhesive, and oxidation wear, alongside a decline in surface quality due to tool-nose passivation after 18 min of cutting. Touggui et al. [16] compared cermet and coated-carbide inserts in turning AISI 316L stainless steel, reporting better surface quality and lower cutting forces with coated carbide but longer tool life (1.25×) with cermet. Zhao et al. [17] examined a Ti(C,N)–TiB_2_–Co cermet in high-speed dry turning of AISI 304, finding lower roughness, shallower hardened layers, and superior adhesive wear resistance compared with commercial carbides. Reis et al. [18] investigated coated cermet and multilayer-coated carbide tools in dry turning AISI 4340, noting lower flank and crater wear in cermets, with adhesive and abrasive mechanisms dominating.

Sarjana et al. [19] evaluated uncoated and PVD-coated TiCN-based cermet tools in turning hardened AISI 4340 (50 HRC). Both tools performed well in finishing operations, with uncoated cermets offering higher productivity and coated variants providing better surface quality. Flank wear was the dominant wear mode, with chipping occurring beyond VBB ≈ 125 μm (uncoated) and 100 μm (coated). Magalhães et al. [20] studied dry turning of AISI 1045 with uncoated cermets, confirming the viability of the process. A tool life of 35 min was reported at 175 m/min (VBB = 0.1 mm), decreasing to 23 min at 275 m/min, with abrasive wear predominating and no detrimental microstructural alterations observed. Das et al. [21] found lower cutting forces and tool wear with cermet inserts compared with uncoated carbides in dry turning of hardened AISI 4340. In dry turning of AISI 304, Sharma et al. [13] reported ~25% lower tool wear, 15% lower Rz, and ~200% longer tool life with coated carbides relative to uncoated tools, recommending multilayer TiAlN/TiN coatings.

Other studies highlight the influence of tool wear on surface roughness, such as notch wear on secondary flanks in hard turning of AISI 5140 with mixed ceramic wipers [22]. Research on alternative cooling strategies, including MQL, cryogenic CO_2_, and high-pressure emulsions, has also yielded promising results [5,23,24,25] though dry turning retains advantages regarding operator health and environmental safety.

In addition to experimental investigations, numerical simulations have increasingly been employed to better understand the fundamental mechanisms involved in machining processes. Techniques such as finite element modeling (FEM), molecular dynamics (MD), and smoothed particle hydrodynamics (SPH) have been applied to study chip formation, temperature evolution, and material removal mechanisms at different scales. For instance, Chen et al. [15] investigated tool wear mechanisms during dry cutting of AISI 304 stainless steel using experimental observations combined with microstructural and compositional analyses, highlighting the roles of adhesion, abrasion, and oxidation in tool degradation. In parallel, numerical approaches have been used to investigate cutting mechanisms that are difficult to observe experimentally. He et al. [26] investigated the influence of temperature on the ductile-to-brittle transition during diamond cutting using an SPH model and demonstrated that increasing temperature promotes ductile deformation due to thermal softening effects. These numerical approaches provide valuable insight into cutting mechanics and material behavior that are difficult to observe experimentally. However, despite advances in computational modeling, experimental studies remain essential for validating machining performance under realistic cutting conditions and for evaluating practical indicators such as tool wear and surface integrity.

Although numerous studies have investigated the machining of stainless steels using different tool materials and cooling strategies, the dry turning of austenitic stainless steels using uncoated cermet inserts remains insufficiently explored, particularly with respect to the combined evaluation of tool wear mechanisms and three-dimensional surface characterization. Previous investigations have often focused either on tool life or surface roughness using conventional 2D profilometry. Furthermore, while numerical studies have provided valuable insights into the fundamental mechanisms of machining processes, experimental validation under industrially relevant cutting conditions remains necessary to assess the practical performance of cutting tools and the resulting surface integrity.

In this context, the present study investigates the dry turning of AISI 304 stainless steel using uncoated cermet inserts under different cutting speeds and feed rates. Unlike many previous studies that relied primarily on conventional two-dimensional roughness parameters, this work combines tool wear analysis with three-dimensional surface metrology in order to provide a more comprehensive characterization of the surface integrity generated during machining. Particular attention is given to the relationship between tool wear evolution and the resulting surface topography under fully dry cutting conditions. By integrating flank wear evaluation, surface roughness measurements, and 3D surface texture analysis, this study aims to clarify the feasibility of using modern cermet tools for sustainable dry turning of stainless steels while maintaining acceptable tool life and surface quality. Therefore, the novelty of this work lies in the combined experimental evaluation of tool wear mechanisms and three-dimensional surface topography during the dry turning of AISI 304 using uncoated cermet inserts, providing new insight into the relationship between tool degradation and surface integrity under sustainable machining conditions.

## 2. Materials and Methods

### 2.1. Experimental Procedure

External cylindrical turning under dry conditions was carried out on AISI 304 stainless steel round bars (30 mm in diameter and 55 mm in length). According to the manufacturer’s datasheet, the chemical composition of the alloy (wt%) is 68.80 Fe, 19.28 Cr, 11.25 Ni, 0.03 C, 0.028 S, 1.73 Mn, 0.030 P, 0.54 Si, and 0.04 N. The machining tests were conducted using a Boxford^®^ CNC lathe (model 160 VMCi, Halifax, UK) with a maximum spindle speed of 3200 rpm and a power of 0.5 kW. The material was machined in the as-received condition, exhibiting a hardness of 215 HB. Additional mechanical properties are presented in Table 1. The workpieces were secured using a three-jaw pneumatic chuck.

**Figure 1:**
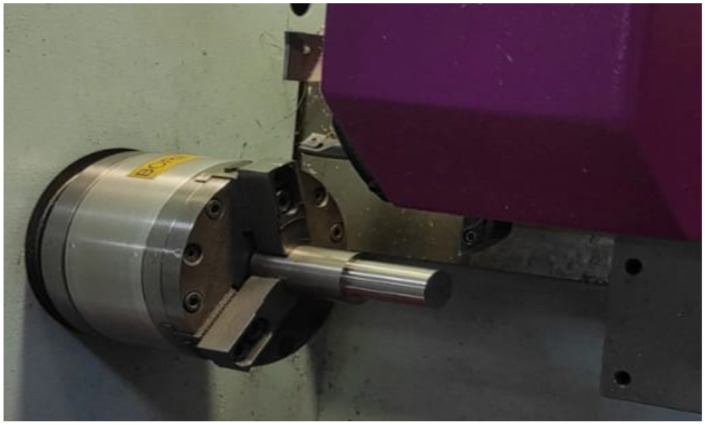
Experimental setup showing the workpiece and tool configuration.

Uncoated cermet inserts (grade T1200A) with DNMG090202N-SC geometry and a positive rake angle of 6° were mounted on an SDACR062B tool holder, both supplied by Sumitomo Tools (Osaka, Japan), as illustrated in Figure 1. According to the manufacturer, the insert microstructure consists of a tough composite phase composed of coarse grains, a W-rich hard phase, and fine TiCN grains dispersed within the binder phase.

Two cutting speeds (Vc) were investigated: 120 m/min and 150 m/min, representing the lower and higher levels, respectively. Feed rate (f) was also varied at two levels, 0.025 mm/rev and 0.05 mm/rev. The depth of cut (doc) was maintained constant at 0.2 mm, corresponding to 100% of the tool corner radius. This value was selected to produce relatively small chip widths and, consequently, lower cutting forces. The complete set of machining parameters is summarized in Table 2. Prior to each experiment, a preliminary pass was performed using a dedicated tool in order to ensure a uniform surface condition. Each cutting condition was repeated twice using a new cutting edge in order to ensure repeatability of the experiments. The variability between repetitions was evaluated, and the divergence between measurements remained below 5% for all tested conditions, indicating good experimental consistency.

### 2.2. Surface Finish

**Figure 2:**
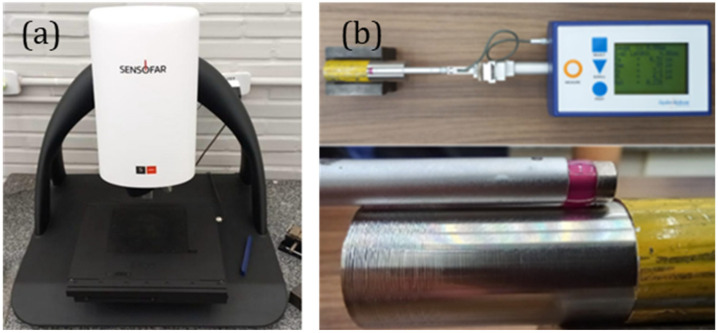
Surface roughness evaluation: 3D non-contact (**a**) and 2D contact (**b**).

First, surface roughness was measured using a Taylor Hobson Surtronic 25 contact profilometer (Leicester, UK), Figure 2a. A cut-off length of 0.25 mm was selected in accordance with ISO 4288 [27]. For each sample, the reported roughness values represent the average of three measurements.

In the second stage, the surface topography of the samples machined with new inserts was assessed using white-light interferometry, which provides enhanced visualization and characterization of surface integrity. Figure 2b. A Sensofar S Neox 3D optical profilometer (Terrassa, Spain) was used. This equipment combines interferometry, confocal microscopy, and focus-variation techniques. The scanning was performed at a rate of 100 µm/s, following the procedures specified in ISO 25178 [28].

### 2.3. Tool Life and Tool Wear

Tool life experiments were performed using an end-of-life criterion defined by an average flank wear (VBB) of 0.1 mm, following the recommendations of ISO 3685 [29]. For each machining condition, the reported results correspond to the mean value obtained from three measurements. The evolution of tool wear was evaluated at intervals equivalent to 150 mm of machined length.

Flank wear measurements were carried out using a Carl Zeiss Discovery V12 stereomicroscope (Oberkochen, Germany) coupled with an AxioCam 305 camera. Image acquisition and analysis were performed using AxioVision V4.7 software (2008, Jena, Germany). The wear mechanisms were further analyzed by scanning electron microscopy (SEM) using a JEOL JSM-6610LV microscope (Tokyo, Japan) equipped with a Bruker X-Flash energy-dispersive X-ray spectroscopy (EDS) detector (Billerica, MA, USA).

## 3. Results and Discussion

### 3.1. Surface Roughness

**Figure 3:**
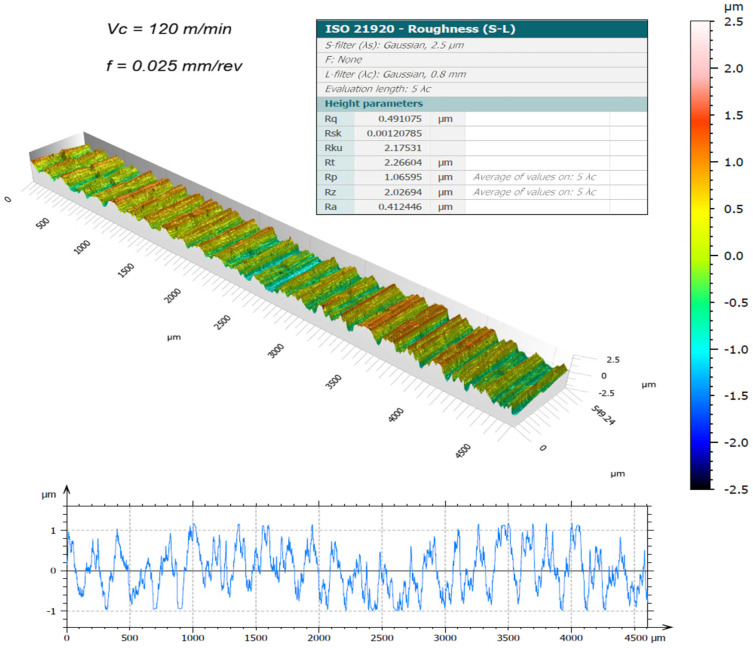
3D surface roughness for Vc = 120 m/min and f = 0.025 mm/rev [30].

**Figure 4:**
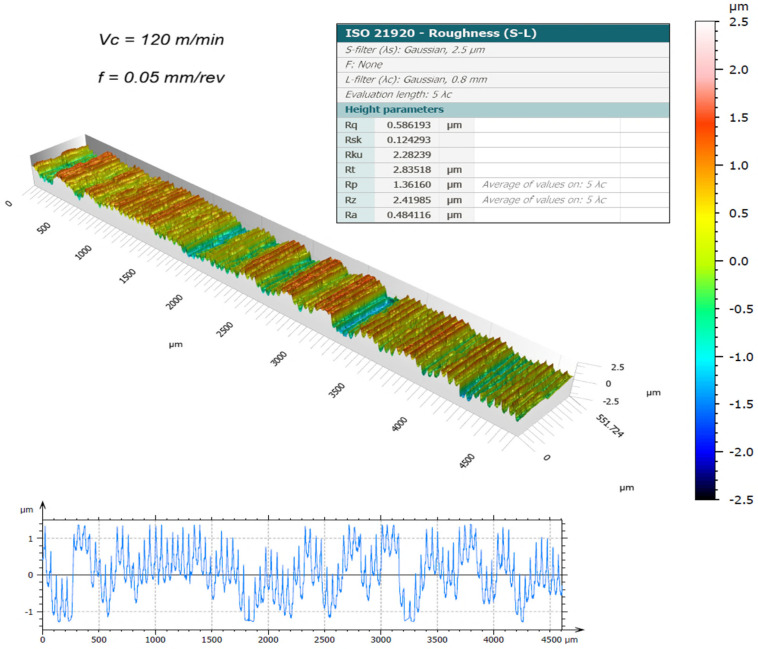
3D surface roughness for Vc = 120 m/min and f = 0.05 mm/rev [30].

**Figure 5:**
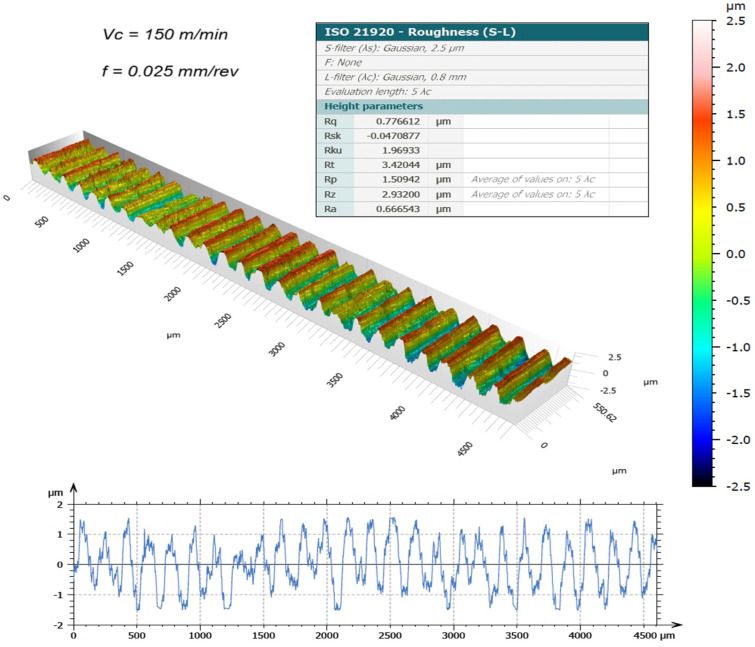
3D surface roughness for Vc = 150 m/min and f = 0.025 mm/rev [30].

**Figure 6:**
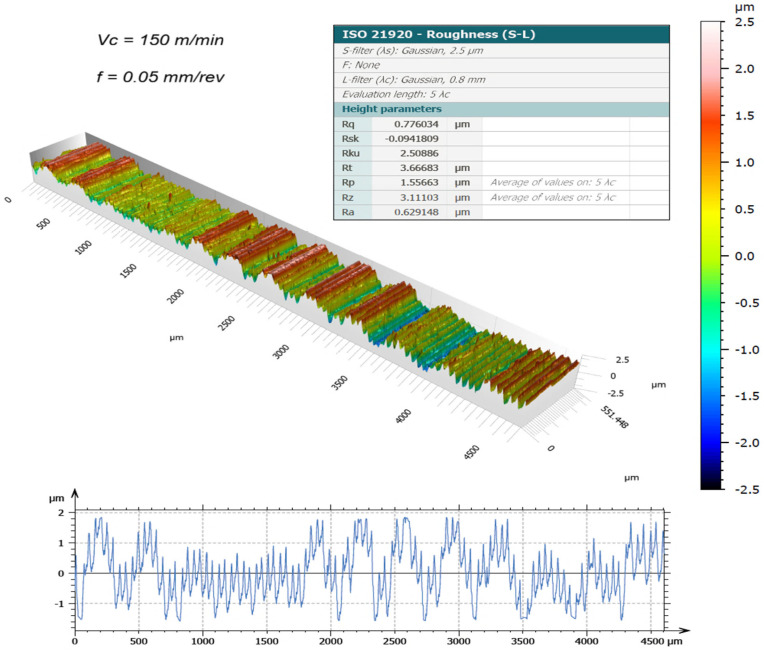
3D surface roughness for Vc = 150 m/min and f = 0.05 mm/rev [30].

**Figure 7:**
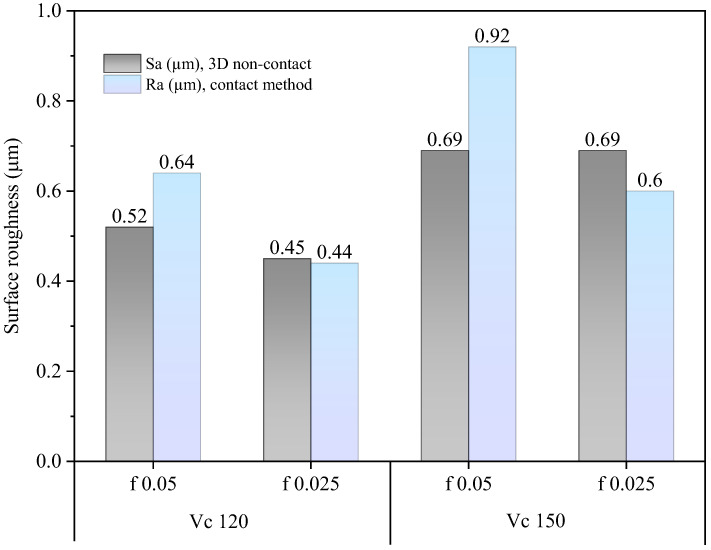
Surface roughness results data (Ra and Sa) by contact and 3D non-contact method.

The surface topography of the machined samples, obtained using a new cutting edge and measured by the 3D optical profilometer, is presented in Figure 3, Figure 4, Figure 5 and Figure 6. The corresponding surface-roughness results obtained with the contact profilometer are summarized in Figure 7.

Based on the contact measurements, at a cutting speed of 120 m/min, the roughness ranged from Ra = 0.44 µm (0.025 mm/rev) to Ra = 0.64 µm (0.05 mm/rev). At 150 m/min, the roughness increased, ranging from Ra = 0.59 µm to Ra = 0.92 µm, respectively. The variability between repeated measurements was low, remaining within approximately 5%, which indicates good repeatability of the experimental procedure and reliability of the reported roughness values. These results indicate that, for the machining conditions investigated, increasing the cutting speed led to a deterioration in surface finish. This behavior can be associated with the higher temperatures generated in the cutting zone during dry machining, since the absence of coolant limits heat dissipation and promotes heat concentration at the tool–chip interface. Austenitic stainless steels such as AISI 304 are well known for their pronounced work-hardening tendency and relatively low thermal conductivity, which further contributes to the accumulation of heat during cutting. Under elevated temperatures and severe plastic deformation, the near-surface layer of the material undergoes significant strain hardening, becoming harder and more resistant to shearing. This condition increases the frictional interaction between the chip and the tool rake face, which may promote unstable chip formation and intensify adhesion phenomena at the tool–chip interface. As a consequence, higher mechanical and thermal loads act on the cutting edge, accelerating the progression of tool wear and altering the effective tool geometry. The degradation of the cutting edge and the increased friction during chip flow ultimately contribute to the formation of more pronounced surface irregularities, leading to higher roughness values on the machined surface. Similar trends have been reported in dry turning studies of stainless steels, where increasing cutting speed resulted in higher temperatures, intensified tool wear mechanisms, and deterioration of surface quality, as discussed by Sharma et al. [13] and Chen et al. [15].

The benefits of three-dimensional surface characterization are evident, as errors associated with stylus–surface contact are eliminated and a full area, rather than a single profile, is evaluated. This allows a more representative characterization of the functional surface texture generated during machining. In conventional 2D profilometry, isolated peaks or valleys may disproportionately influence the measured roughness values. In contrast, the areal parameters obtained by interferometry provide a more robust description of the surface morphology produced by the turning process. The lower Sa values compared with Ra observed in this study suggest that localized irregularities along individual measurement traces may have influenced the contact measurements. For example, at 150 m/min with the highest feed rate (0.05 mm/rev), the 3D method yielded Sa = 0.69 µm, whereas the contact method produced Ra = 0.92 µm. Similar trends were observed for the Rz and Sz parameters.

It is worth highlighting that the Sa values were generally lower than the corresponding Ra values. This difference can be attributed to the distinct nature of profile-based and areal surface characterization methods. Conventional 2D profilometry evaluates roughness along a single sampling line, which may capture isolated peaks or valleys and therefore overestimate the average roughness. In contrast, the Sa parameter represents the arithmetic mean height over a measured area, providing a more statistically representative description of the surface. In addition, turning typically produces anisotropic surface textures characterized by periodic feed marks. Depending on the orientation of the stylus path relative to these features, the Ra value may be influenced by localized surface irregularities. Areal measurements obtained by interferometry reduce this directional bias and therefore often yield slightly lower roughness values compared with profile measurements.

The Sa parameter is the areal analogue of Ra, representing the arithmetic mean height of the surface Z(x,y) over the evaluation area. It provides stable and robust results since it is less affected by scratches, contaminants, and measurement noise. The Sz parameter is the areal counterpart of Rz; it represents the maximum height of the surface and is defined as the sum of the maximum peak height (Sp) and the maximum valley depth (Sv) [31].

Overall, the results demonstrate that excellent surface finish quality can be achieved under dry machining conditions. The interferometry analyses confirmed that, when the cutting edge remained in acceptable condition, the machined surfaces were free of defects and exhibited uniform integrity. The deterioration of surface quality observed at higher cutting speeds was closely associated with the evolution of tool wear. As flank wear and edge chipping increased, the effective tool geometry changed, promoting unstable cutting conditions and contributing to the observed increase in roughness parameters.

### 3.2. Tool Wear

**Figure 8:**
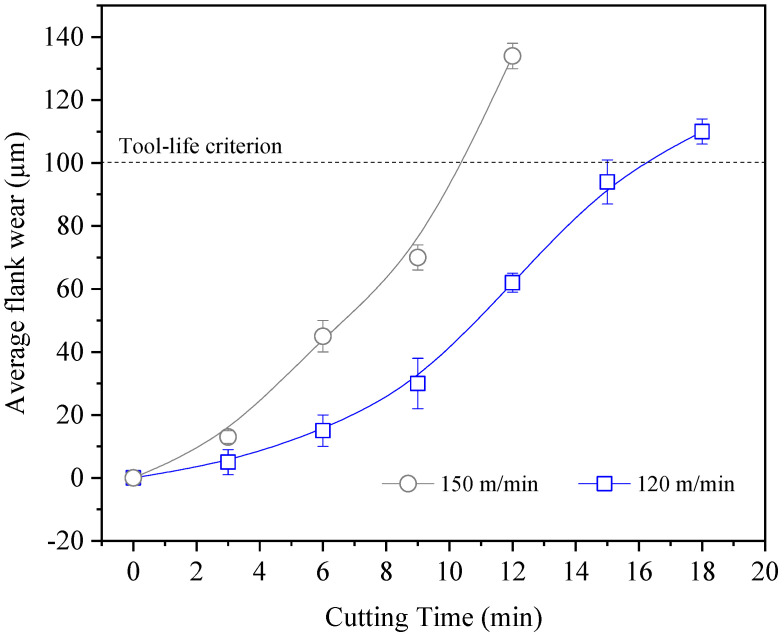
Average flank wear VB_B_, according ISO 3685.

The average flank-wear (VBB) curves, obtained in accordance with ISO 3685 and using 100 µm as the failure criterion, are presented in Figure 8. At a cutting speed of 120 m/min, the tool life was approximately 18 min, whereas at 150 m/min it was reduced to about 10 min.

**Figure 9:**
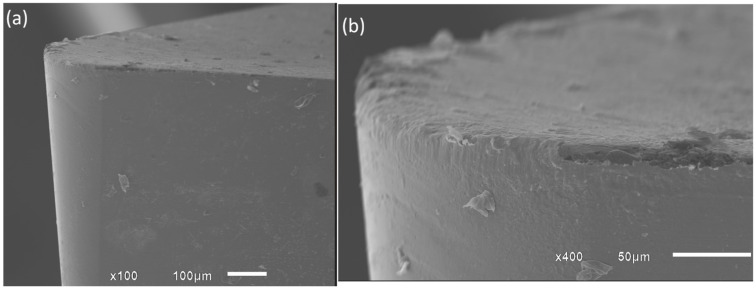
Tool-tip and flank wear morphology at the end-of-life criterion for Vc = 120 m/min, observed under: (**a**) 100× magnification and (**b**) 400× magnification.

**Figure 10:**
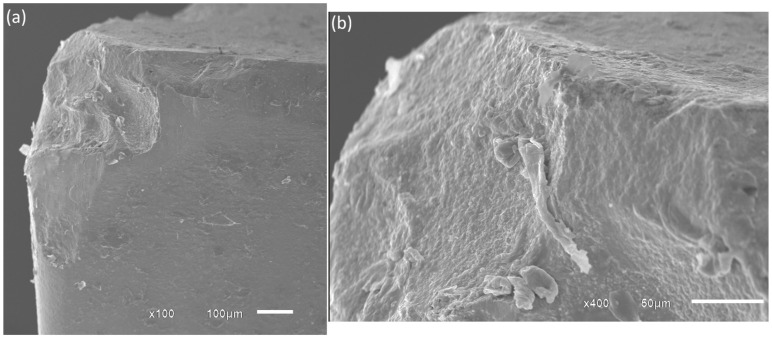
Tool-tip wear at the tool life criterion for Vc = 150 m/min: (**a**) 100× magnification; (**b**) 400× magnification.

**Figure 11:**
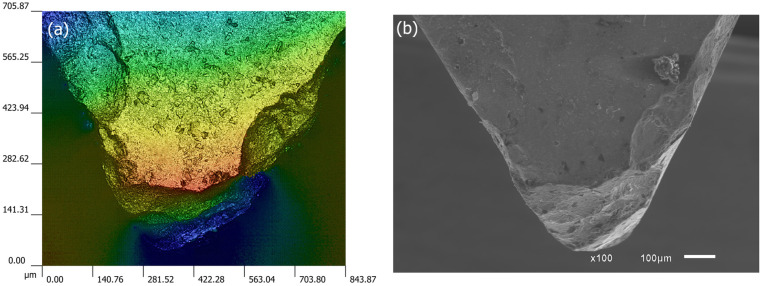
Tool-tip condition at the tool life criterion for Vc = 150 m/min using complementary techniques: (**a**) Focus variation, 100× magnification; (**b**) SEM.

**Figure 12:**
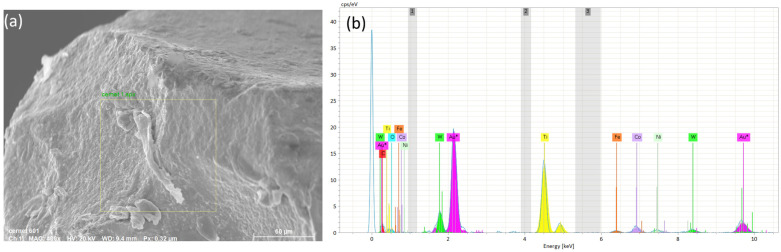
SEM (**a**) and EDS analysis (**b**) of the tool-tip region at the tool life criterion for Vc = 150 m/min.

SEM images of the tool tip and main flank at the moment the failure criterion was reached are shown in Figure 9, Figure 10, Figure 11 and Figure 12. These analyses were conducted using the lower feed rate (0.025 mm/rev). Figure 9 shows the insert used at a cutting speed of 120 m/min. It can be observed that abrasive wear is the predominant mechanism (Figure 9a), with adhesion and minor chipping also present. The tool tip largely maintains its integrity. Pronounced wear can also be seen on the secondary flank (Figure 9b). This behavior is expected because this region of the tool remains in continuous contact with the newly machined surface, where friction and sliding between the tool flank and workpiece generate additional thermal and mechanical loads. These conditions promote abrasive wear and material adhesion, contributing to the progressive enlargement of the flank wear land [32].

Figure 10 shows the insert used at a cutting speed of 150 m/min. At this speed, chipping and adhesion were the predominant wear mechanisms. The occurrence of chipping is likely related to the higher mechanical and thermal loads acting on the cutting edge at 150 m/min. Under dry machining conditions, the absence of coolant limits heat dissipation, causing a significant increase in temperature at the tool–chip interface. This thermal load may reduce the fracture resistance of the cermet edge and promote micro-fracture propagation, ultimately resulting in chipping. In addition, the strong chemical affinity between austenitic stainless steels and tool materials promotes adhesion and the formation of built-up material on the tool surface, which further destabilizes the cutting edge.

Figure 11 presents the condition of the tool tip and rake face using two complementary techniques: (Figure 11a) focus variation and (Figure 11b) SEM. Chipping propagated into both the rake face and the secondary flank, further contributing to the degradation of surface quality. The tool tip no longer maintained its structural integrity, and significant adhered material can also be observed (Figure 11b).

EDS analysis (Figure 12b) of a selected area (Figure 12a) on the main flank of the insert indicate a possible diffusion of nickel in the worn region of the insert, suggesting material transfer from the workpiece to the tool surface during cutting. This phenomenon is typical of diffusion-assisted wear mechanisms occurring at elevated temperatures. The presence of adhered or diffused elements from the workpiece confirms the strong adhesive interaction between the AISI 304 stainless steel and the cermet tool under dry machining conditions. In contrast, chromium appears to remain largely unaffected. Additionally, the W-rich tough hard phase and the fine TiCN grain phase seem to retain their structural integrity, indicating that the primary wear processes were associated mainly with adhesion and diffusion phenomena rather than extensive degradation of the cermet matrix.

Considering the surface finish results, they can be regarded as quite satisfactory when compared with previous studies in the literature [13] when uncoated cermets were used, particularly because lower feed values were employed in the present work. The surface finish achieved with a new cutting edge is comparable to the results reported by Sampaio et al. [5] under both dry and MQL conditions.

The observed wear mechanisms are consistent with those reported in previous studies on dry turning of stainless steels, where adhesion, abrasion, and diffusion wear are commonly observed due to the combination of high cutting temperatures and strong chemical affinity between the workpiece material and the tool [14,15]. The occurrence of chipping at relatively moderate cutting speeds in the present work suggests that the thermal load associated with dry machining of AISI 304 may significantly influence the stability of the cutting edge. However, it is important to note that each study is influenced by specific experimental conditions, including cutting parameters, tool geometry, and equipment. In this work, the cutting speed of 120 m/min provided a more stable machining condition under dry turning. At this speed, tool wear progressed more gradually and the resulting surface finish remained within acceptable levels. In contrast, increasing the cutting speed to 150 m/min intensified thermo-mechanical loads at the tool–chip interface, promoting edge chipping and contributing to the deterioration of surface roughness. From a sustainability perspective, operating at 120 m/min may represent a more favorable condition, since the improved stability of the cutting edge reduces premature tool replacement and supports consistent surface quality without the use of cutting fluids. Although higher cutting speeds may increase productivity by raising the material removal rate, the more moderate cutting speed investigated in this study provides a more sustainable compromise between machining efficiency, tool life, and surface integrity, while avoiding the environmental and operational impacts associated with coolant systems.

For future work, it is planned to explore the use of MQL and coated cermet tools to evaluate their performance and compare the results with those obtained in the present study.

## 4. Conclusions

The main conclusions of this work are as follows:

✓ Influence of cutting parameters on surface roughness: Both feed and cutting speed significantly affected surface roughness. As expected, increasing the feed worsened the surface finish due to the larger feed marks generated during turning. Additionally, higher cutting speeds promoted a gradual increase in surface roughness. The best surface quality was obtained at the lowest feed and cutting speed investigated, achieving Sa ≈ 0.45 µm with a new cutting edge. Overall, roughness values remained below approximately Ra ≈ 1.0 µm (Sa ≈ 0.45–0.70 µm), which corresponds to typical requirements for fine finishing operations.
✓ Tool life and wear mechanisms: At a cutting speed of 120 m/min, the tool life was approximately 18 min, whereas at 150 m/min it decreased to around 10 min. At the lower cutting speed, abrasive wear and adhesion were predominant, and the tool tip maintained its integrity until the failure criterion (VBB = 100 µm) was reached. At the higher cutting speed, higher thermo-mechanical loads promoted adhesion, diffusion, and edge chipping, accelerating tool degradation.
✓ Industrial and sustainability implications: The results demonstrate that dry turning of AISI 304 using uncoated cermet inserts is feasible for finishing operations. For the conditions investigated, a cutting speed of 120 m/min combined with low feed rates (≈0.025 mm/rev) provided the best compromise between surface quality, tool life, and process stability. In addition to improving tool durability, this parameter range supports sustainable machining by eliminating cutting fluids and reducing the environmental and operational costs associated with coolant systems.

Overall, the study confirms that modern cermet tools can successfully enable sustainable dry finishing of AISI 304 stainless steel when moderate cutting speeds and low feeds are applied. These findings provide practical guidance for industrial applications and support further research on optimized dry machining strategies, including the use of coated cermets or hybrid lubrication techniques such as MQL.

## Figures and Tables

**Table 1:** Material properties.

Properties	Values
Tensile strength (MPa)	518
Yield strength (MPa)	310
Hardness (HB)	215

**Table 2:** Test cutting parameters.

Vc (m/min)	f (mm/rev)	Doc (mm)
120	0.025	0.2
	0.05	
150	0.025	
	0.05	

## Data Availability

The original contributions presented in this study are included in the article. Further inquiries can be directed to the corresponding authors.
